# Biopsychosocial experiences of Chinese university dancers during injury rehabilitation: a qualitative study

**DOI:** 10.3389/fpsyg.2026.1856362

**Published:** 2026-06-24

**Authors:** XiaoWen Gao, Hanbyul Kim, Moonjung Bae

**Affiliations:** 1 College of Music and Dance, Hebei Minzu Normal University, Chengde, China; 2 Department of Physical Education, Korea National Sport University, Seoul, Republic of Korea; 3 H+ Yangji Hospital, Seoul, Republic of Korea

**Keywords:** athletic injuries, biopsychosocial perspective, dancing, qualitative research, rehabilitation

## Abstract

Injury rehabilitation is a complex process shaped by interacting biological, psychological, and social factors. This study aimed to explore the biopsychosocial experiences of Chinese university dancers during injury rehabilitation. A qualitative study was conducted using semi-structured in-depth interviews with seven Chinese university dancers who had experienced injury rehabilitation. The data were analyzed using thematic analysis to identify key themes across biopsychosocial domains. The findings revealed that injury rehabilitation experiences were multidimensional. At the biological level, participants reported physical symptoms such as pain, joint instability, and reduced flexibility, along with somatic responses including headaches and sleep disturbances. Some dancers also described physical growth following rehabilitation, including improved strength and flexibility. At the psychological level, negative emotions such as anxiety, frustration, anger, and guilt were prominent, while resilience grounded in a positive mindset also emerged. At the social level, emotional and practical support from parents, teachers, and peers functioned as protective factors, whereas pressure to return, indifference, and criticism acted as risk factors. Notably, participants’ experiences were situated within a broader cultural context, which influenced their perceptions of injury, responses to rehabilitation, and interactions with their social environment. These findings suggest that dancers’ injury rehabilitation should be understood within an expanded biopsychosocial framework that incorporates cultural context. The study highlights the need for integrated and culturally sensitive rehabilitation interventions for injured dancers. Future research should adopt cross-cultural and longitudinal approaches to further elucidate these experiences.

## Introduction

Dance is a high-intensity physical activity that requires both artistic expression and advanced physical performance, and injuries frequently occur during repetitive training and performance processes (Sohl and Bowling, 1990). Previous studies have reported a high prevalence of musculoskeletal injuries among dancers, particularly involving the lumbar spine and lower extremities (Smith et al., 2015; Sun and Liu, 2024).

Chinese dance, which requires technically demanding movements involving flexibility, balance, muscular strength, and expressive performance, also presents a high risk of injury. A prospective study of university-level Chinese dancers reported an injury incidence of 3.28 injuries per 1,000 dance hours, with the low back, knee, and ankle being the most commonly affected sites (Xue et al., 2023). In addition, chronic ankle instability has been reported among Chinese traditional dancers and was associated with pain and reduced quality of life (Chui et al., 2022). Given the high risk of injury among dancers, systematic management and targeted rehabilitation approaches are needed.

Injuries in dancers are not confined to physical damage alone. Injured dancers frequently report a range of psychological difficulties, including career-related anxiety, self-doubt, performance pressure, and perfectionism (Dong et al., 2025). Contemporary dancers who have experienced injuries have been shown to undergo negative emotional responses such as disappointment, sadness, frustration, anger, and anxiety. They also report adverse cognitive reactions, including distrust in the recovery process and a perceived loss of dancer identity (Fernandez et al., 2023). Taken together, injuries can elicit substantial negative emotional and cognitive responses in dancers. These responses may be particularly pronounced among Chinese university dancers, who are often exposed to highly competitive environments, potentially intensifying maladaptive reactions following injury.

Psychological factors that arise following injury play a critical role in rehabilitation outcomes. Variables such as injury-related and performance-related anxiety, confidence, and motivation have been reported to significantly influence the success of rehabilitation (Ardern et al., 2013; Forsdyke et al., 2016). In addition, athletes’ perceptions of the legitimacy of rehabilitation training have been shown to affect rehabilitation adherence and outcomes (Goddard et al., 2021). Among dancers, key psychological factors influencing both injury occurrence and rehabilitation outcomes include stress, psychological distress, eating disorders, and coping strategies (Mainwaring and Finney, 2017).

Social factors also play a crucial role in injury rehabilitation outcomes. Key social influences include tangible support, emotional support, and informational support. Tangible support refers to the direct provision of financial resources or material assistance necessary for rehabilitation, whereas emotional support involves expressions of care, encouragement, and reassurance from significant others. Informational support encompasses the provision of knowledge related to injury recovery or recommendations of relevant professionals and resources. Such forms of social support have been shown to alleviate negative psychological responses in injured athletes (Yang et al., 2014; Forsdyke et al., 2022), which may, in turn, contribute positively to successful rehabilitation outcomes.

Taken together, it is essential to consider biological, psychological, and social factors when addressing injury rehabilitation. A biopsychosocial approach to injury rehabilitation has been shown to enhance rehabilitation outcomes and facilitate a more effective return to performance (Brewer and Redmond, 2016). In particular, recent research has increasingly emphasized that understanding and supporting psychological and social experiences, alongside biological factors, are critical to injury rehabilitation and substantially influence rehabilitation outcomes (Bae, 2024). Therefore, identifying the biopsychosocial experiences of dancers during injury rehabilitation may contribute to improving rehabilitation outcomes.

Despite this, previous research on injuries among Chinese university dancers has predominantly consisted of quantitative studies focusing on biological aspects, such as injury epidemiology and risk factors (Chui et al., 2022; Lai et al., 2022; Hung et al., 2023; Dang et al., 2024). In contrast, there is a relative paucity of qualitative research that comprehensively and in depth explores the biopsychosocial experiences of Chinese university dancers during injury rehabilitation. Accordingly, the present study aimed to explore the experiences of Chinese university dancers during injury rehabilitation from a biopsychosocial perspective. Specifically, this study sought to examine the biological, psychological, and social experiences encountered during injury rehabilitation, as well as how these dimensions interact and influence rehabilitation outcomes. This study is expected to advance understanding of the multifaceted challenges faced by injured Chinese dancers and to provide a foundation for the development of rehabilitation programs that integrate physical, psychological, and social dimensions.

## Materials and methods

### Participants

This study employed in-depth interviews to explore the biological, psychological, and social experiences of Chinese university dancers during injury rehabilitation. A total of seven Chinese university dancers with prior injury rehabilitation experience participated in the study. The detailed inclusion and exclusion criteria are presented in Table 1. Participants were eligible if they were university students majoring in Chinese dance who had undergone at least 4 weeks of rehabilitation due to injury and had been unable to participate in training, performances, or competitions for a minimum of 4 weeks. Individuals who declined to participate in the study were excluded.

**TABLE 1:** Inclusion and exclusion criteria for participants.

Criteria type	Eligibility criteria
Inclusion criteria	- Chinese university students majoring in dance who have experienced an injury - Chinese university dance majors who have been unable to participate in training, performances, or competitions for at least 4 weeks due to injury - Chinese university dance majors who have undergone rehabilitation for at least 4 weeks due to injury
Exclusion criteria	- Chinese university dance majors who declined to participate in the study

This study adopted a qualitative research design aimed at exploring the specific experiences of Chinese university dancers with injury rehabilitation experience rather than generalizing the findings to a broader population. In small-scale qualitative studies, thematic analysis typically involves conducting in-depth interviews with approximately 6–10 participants (Braun and Clarke, 2013), with recruitment continuing until no new information emerges and data saturation is achieved. Accordingly, in the present study, in-depth interviews were conducted with seven participants, a number deemed sufficient to obtain rich and relevant data. Data collection proceeded until no new themes emerged and saturation was reached.

Participants were recruited through visits by the researcher to university dance departments in China. The purpose of the study was explained to potential participants, and those who met the inclusion and exclusion criteria and agreed to participate were enrolled in the study. Subsequently, a snowball sampling strategy was employed, whereby enrolled participants referred other dancers who met the eligibility criteria. Through this process, a total of seven participants were recruited. To ensure anonymity, each participant was assigned a unique identifier (S001–S007). None of the participants reported chronic illnesses, disabilities, psychiatric treatment, or medication use, including antidepressants, at the time of the interviews. The demographic characteristics and injury-related information of the participants are presented in Table 2.

**TABLE 2:** Participant characteristics.

Participants	Age	Sex	Dance genre	Dance experience	Injury site	Diagnosis	Surgical history	Rehabilitation duration
S001	19 years	F	Chinese dance	4 years	Clavicle	Fracture	O	3 years
S002	20 years	F	Chinese dance	9 years	Knee	Patellar tendinitis	X	4 years
S003	21 years	F	Chinese dance	6 years	Sacroiliac joint	Sacroiliac joint effusion	X	3 months
S004	20 years	M	Chinese dance	5 years	Lumbar spine	Lumbar disk herniation	X	2 months
S005	20 years	F	Chinese dance	10 years	Cervical spine	Cervical sprain	X	3 months
S006	21 years	F	Chinese dance	10 years	Lumbar spine	Lumbar disc herniation	X	4 years
S007	19 years	F	Chinese dance	10 years	Brain, cervical spine	Concussion, cervical spine trauma	X	1 month

This study was conducted in accordance with the Declaration of Helsinki. Prior to participation, all participants were fully informed about the purpose and procedures of the study, as well as their rights and potential benefits, and provided written informed consent. The study protocol was reviewed and approved by the Institutional Review Board of Korea National Sport University (approval number: 20251119-158). The researcher completed formal training in research ethics prior to data collection.

### Data collection

This study conducted in-depth interviews to explore the biological, psychological, and social experiences of Chinese university dancers during injury rehabilitation. Prior to the interviews, relevant literature was reviewed, and an interview guide was developed through expert consultation, involving two specialists in sports psychology and one specialist in sports medicine.

The in-depth interviews were conducted in a semi-structured format based on the interview guide and took place in a private setting to ensure confidentiality. Each interview began with open-ended questions, followed by probing questions to further explore participants’ responses in depth. All interviews were conducted by the researcher XW, who holds a doctoral degree in sports psychology and has a background in Chinese dance. XW possesses both firsthand experience and professional expertise in dance performance environments and injury-related cultural contexts. This insider position facilitated rapport with participants and contributed to the depth and richness of the interviews. In particular, it enabled a nuanced understanding of issues such as training pressure, hierarchical relationships, and the stigma surrounding injury among Chinese university dancers.

However, the researcher’s insider position may introduce the possibility that prior beliefs and experiences could influence the processes of data collection and interpretation. To minimize this potential bias, the researcher maintained a non-judgmental stance during the interviews, ensuring that participants could freely express their views, and clearly informed them that their responses would not be related to academic evaluation or assessment. In addition, reflexive memos were continuously recorded throughout the interview and analysis processes to monitor the researcher’s preconceptions and emotional responses. Regular discussions with experts in sports psychology (HB) and sports medicine (MJ) were also conducted to enhance the balance and validity of the interpretations. The open-ended questions were as follows:

- Please describe your biological experiences during injury rehabilitation. Biological experiences refer to any physical symptoms or bodily changes, such as pain or muscle weakness.
- Please describe your psychological experiences during injury rehabilitation. Psychological experiences refer to emotions and thoughts, including depression, anxiety, and confidence.
- Please describe your social experiences during injury rehabilitation. Social experiences refer to interactions with significant others, such as parents, coaches, and peers, including forms of tangible and emotional support.

Each participant was interviewed once, with interviews lasting approximately 1 h. With participants’ consent, all interviews were audio-recorded using a portable recording device. Following the interviews, any incomplete or unclear information was supplemented through follow-up contact with participants. To enhance the credibility of the data, expert meetings were conducted throughout the development of the interview guide, the in-depth interview process, and the data analysis. These meetings involved two sports psychologists (XW, HB) and one sports medicine specialist (MJ). At each stage, iterative review and refinement were undertaken until consensus was reached among the three experts, thereby strengthening the reliability of the study’s interpretations and findings. In addition, the results of the data analysis and their interpretations were shared with participants, and their feedback was incorporated to verify the accuracy and authenticity of the data.

### Data analysis

Data were analyzed using thematic analysis as outlined by Braun and Clarke (2006). First, all interviews were transcribed verbatim using Microsoft Word. Interviews were conducted and recorded in Chinese, the native language of the participants. The interview data and quotations included in the manuscript were translated into English by researcher XW, who is a native Chinese speaker with expertise in Chinese dance and sport psychology and is fluent in English. To preserve contextual and semantic accuracy, repeated reviews were conducted throughout the translation process, and the translated materials were additionally reviewed by a colleague fluent in English.

The researchers then repeatedly read the transcripts to identify meaningful statements relevant to the research purpose. These statements were subsequently coded using terms that best captured their underlying meanings. The generated codes were grouped based on conceptual similarity and organized into categories, from which themes were developed. The identified themes were then reviewed to ensure that they accurately reflected the original data, followed by refinement and revision where necessary. Each theme was clearly defined and assigned a representative name that captured its core meaning. Finally, the confirmed themes were used to describe the biopsychosocial experiences of dancers during injury rehabilitation, and direct quotations from participants were included to support each theme. A detailed example of the data analysis process is presented in Table 3.

**TABLE 3:** Example of the thematic analysis process.

Participant	Significant statement	Formulated meaning	Subtheme	Theme	Category
S001	I felt worried and afraid that I might no longer be able to dance.	I was worried and afraid that I might no longer be able to dance.	Anxiety about returning	Anxiety	Psychological experiences
S001	I was also concerned about what my parents might say. I worried that even after recovery, they might consider dancing too risky and prevent me from dancing again.	I was concerned that my parents might stop me from dancing because they believed it was dangerous.	Anxiety about returning	Anxiety	Psychological experiences
S001	During video calls, my friends consistently shared positive messages and offered strong encouragement and comfort. Online friends also supported me, but the emotional support from my close friends was the most significant.	My friends offered encouragement and comfort during video calls.	Emotional support from peers	Emotional support	Social experiences
S001	–	–	–	–	–

During the data analysis process, an expert team comprising two sports psychologists (XW, HB) and one sports medicine specialist (MJ) conducted cross-validation of the data. In cases where discrepancies in interpretation arose, these were resolved through in-depth discussion until consensus was reached.

## Results

As a result of this study, a total of 11 themes and 40 subthemes were identified, reflecting the biological, psychological, and social experiences of Chinese university dancers during injury rehabilitation. The detailed themes and subthemes for each domain are presented in Table 4.

**TABLE 4:** Biopsychosocial experiences of Chinese university dancers during the injury rehabilitation process.

Category	Theme	Subtheme
Biological experiences	Injury-related physical symptoms	Pain, instability, reduced flexibility, lower limb imbalance, surgical site scarring, concussion-related symptoms
	Somatic symptoms	Headache, insomnia, gastrointestinal symptoms, hair loss
	Physical growth following rehabilitation	Increased muscle strength, improved flexibility
Psychological experiences	Anxiety	Anxiety about falling behind peers, anxiety about returning to performance, anxiety about performance decline, anxiety about lasting effects of injury, anxiety about reinjury
	Frustration	Frustration due to performance decline, frustration due to reinjury
	Anger	Anger about the occurrence of injury, anger due to exclusion from training
	Guilt	Guilt toward dance partners, guilt toward parents
	Resilience	Positive mindset
Social experiences	Emotional support	Parents’ accepting attitude, emotional support from teachers, Emotional support from peers
	Tangible support	Treatment-related tangible support, daily life–related tangible support
	Negative social experiences	Pressure to return, indifference, encouragement to discontinue dancing, criticism

### Category 1. Biological experiences

#### Theme 1. Injury-related physical symptoms

Participants reported a range of injury-related physical symptoms, including pain in the clavicle, cervical spine, lumbar spine, and knee. Pain was relatively severe in the early stages of injury but generally improved over the course of rehabilitation. However, some participants reported recurrent pain during specific movements even as recovery progressed. Participant S006, who experienced a lumbar disk herniation, reported neurological symptoms such as lower limb numbness due to nerve compression. Several participants also experienced instability and reduced flexibility during certain movements, and lower limb imbalance associated with injury-related pain. Participant S001 underwent surgery for a clavicle fracture and subsequently developed a prominent scar at the surgical site. Participant S007 sustained a concussion after falling onto the head during a partnered dance movement, and reported concussion-related symptoms such as headache, vomiting, and nausea.

*“My clavicle fracture was the most painful. It was quite severe at first, but the pain gradually subsided as I recovered. I didn’t experience significant pain in other areas.”(S001)*

*“The injured area was extremely painful, and I couldn’t move at all at first. The ligaments in my right arm also hurt. When I tried to get up, I had to keep my torso straight and lift myself together with my neck. It even affected my sleep—I felt uncomfortable turning from side to side.” (S005)*

*“The injured area was definitely painful. My injury was in the lower part of my back, and it compressed my legs, causing severe numbness. Both of my legs felt numb.” (S006)*

*“There’s a side leg kick in one of the repertoires. When I hold my leg and kick, my knee hurts at the moment I extend it with force, and it sometimes feels unstable. My sense of balance has also worsened. I used to be able to lift my leg to 180 degrees, but now, after injuring both knees, I can’t lift it as high.” (S002)*

*“When I walked, one side was higher and the other lower. I didn’t notice it myself, but…” (S001)*

*“I tend to develop noticeable scars, so after the surgery, the scar became quite large. The scar treatment was very expensive, so I didn’t want my family to buy it, but eventually I developed hypertrophic scarring.” (S001)*

*“It was mainly my head, because I had a brain injury, so I felt dizzy and had headaches. I also had occasional vomiting and nausea. For the first two or three days after the injury, I was in a state of vomiting. When I woke up in the morning, I felt nauseous, but I couldn’t actually vomit—I just felt sick.” (S007)*

#### Theme 2. Somatic symptoms

Participants reported experiencing a range of negative emotional responses during injury rehabilitation, including anxiety and depression. These emotional difficulties were not limited to psychological reactions but were also manifested as physical symptoms in the form of stress-related somatization. Indeed, some participants reported experiencing somatic symptoms such as insomnia, gastrointestinal discomfort, headaches, and hair loss.

*“(After the injury) At first, I was so anxious that I stayed awake all night at the hospital, and even after returning home, I couldn’t sleep. This continued for about 15 days, and I experienced severe hair loss. I also lost my appetite and couldn’t eat anything. I occasionally had headaches, and my stomach pain became much worse than before. I sometimes felt nauseous as well.” (S001)*

*“I had insomnia for a while. Whenever I closed my eyes, I kept thinking about what had happened, and I worried about what I would do if I didn’t recover…” (S003)*

*“I experienced a loss of appetite—I didn’t feel like eating anything…” (S005)*

*“I lost a lot of hair. Every time I showered or dried my hair, it would fall out in clumps.” (S006)*

#### Theme 3. Physical growth following rehabilitation

All participants commonly reported negative physical experiences following injury, such as pain and muscle weakness. However, some participants also described positive physical changes during injury rehabilitation, including improved muscle strength and flexibility during recovery. In this regard, rehabilitation was not perceived merely as a return to baseline function but, in some cases, as an experience of physical growth.

*“After giving my back some rest, I actually became more flexible. My body felt much looser than before, and lifting my back leg became easier.” (S001)*

*“I think my muscles became stronger. Even though I was injured, I had to keep training, so I kept working on both sides of my lower back muscles and my back muscles. As I trained more, they became stronger, which helped protect my lumbar disk to some extent.” (S006)*

### Category 2. Psychological experiences

#### Theme 1. Anxiety

Participants reported experiencing anxiety during injury rehabilitation. In particular, being unable to participate in training led to concerns about falling behind their peers, especially as they observed others continuing to practice. This anxiety appeared to be shaped by the performance-oriented evaluation system emphasized within Chinese dance education.

*“When I had video calls with my friends and heard that they had learned new techniques, I became impatient and envious because I hadn’t learned them yet.” (S001)*

*“When I saw others training, I felt increasingly anxious. I major in Chinese classical dance, and during the final stage of the exam period, seeing the performance results and promotional videos they posted every day made me feel very uneasy and pressured.” (S003)*

*“At that time, I was recovering at home due to the COVID-19 lockdown. Seeing my friends staying at school and improving their skills made me feel very anxious and regretful.” (S004)*

*“When I was in pain, I didn’t want to do back extensions, and I didn’t want my teacher to stretch or open up my back either. But at the same time, I was worried that if I didn’t practice, I would fall behind others.” (S006)*

Participants also reported anxiety regarding their ability to return to dance and resume performance following injury. Some expressed concerns about whether they could regain their previous level of performance after returning. Furthermore, participants reported persistent anxiety about the possibility of reinjury when performing specific movements, even after recovery. This anxiety was not fully resolved following rehabilitation and appeared to continue influencing their overall performance context.

*“I felt worried and afraid that I might not be able to dance anymore…” (S001)*

*“I even thought, ‘What if I can never dance again in the future?”’ (S006)*

*“I felt quite anxious during the entrance exam. I was worried that if I made even one mistake, I might fail completely, and that I wouldn’t be able to perform at my true level…” (S003)*

*“(Before returning) I was worried that I hadn’t fully recovered yet, so I hesitated to try certain movements. I was also concerned that, since I hadn’t practiced for such a long time, my dance skills might have declined compared to others…” (S005)*

*“I was also worried that my performance ability might have decreased…” (S007)*

*“I was worried that my injury might worsen or recur…” (S004)*

*“I definitely have some trauma related to lifting movements, so I was afraid of getting injured again.” (S005)*

*“I still have to perform the movement that caused my injury. Although I was very happy after recovering, I still felt afraid when doing it, worrying that the same situation might happen again. I felt that if it did happen again, it wouldn’t be as simple as before.” (S007)*

Some participants also reported anxiety regarding the potential for long-term sequelae following injury. In particular, participant S007 expressed concerns about whether the concussion might lead to lasting impairments in brain function and whether such effects could result in persistent limitations in their dance performance.

*“I was also worried that my physical condition might deteriorate as I get older. People who have trained in dance often say that there can be long-term aftereffects, don’t they?” (S006)*

*“Right after I got injured, I was extremely scared because the injured area was critical. If there were even slight long-term effects or problems in the back of my head or cervical spine, it could have a major impact on my dance career. So I was very worried and immediately informed my parents, and they were also very concerned. We went to the hospital right away for an examination. The doctor said my condition was unstable and that we needed to monitor my recovery the next day.” (S007)*

#### Theme 2. Frustration

Participants reported experiencing frustration following injury due to declines in performance and the risk of reinjury. This frustration was not merely a transient emotional response but was accompanied by other negative emotions such as anxiety and anger. Some participants described heightened frustration when they were unable to fully regain their previous performance level after rehabilitation or became increasingly aware of the risk of reinjury during performance.

*“At first, I thought it wasn’t a big deal. But after the injury, my knee kept swelling, and I couldn’t perform many movements, which made me feel sad and upset. I also felt regret and disappointment.” (S002)*

*“For a while, I felt better and thought it wasn’t very serious. But after taking a technique class later on, I couldn’t perform again—it completely broke down.” (S003)*

*“After I had recovered, I injured my knee again by chance during a technique class. Experiencing repeated injuries was very frustrating, because my body started hurting again.” (S004)*

#### Theme 3. Anger

Participants reported experiencing anger in response to being excluded from training due to injury. Some participants expressed anger toward the circumstances in which the injury occurred. Participant S007 perceived that the injury resulted from insufficient support by a male partner during a movement and reported experiencing strong anger regarding this situation.

*“I couldn’t regulate my emotions well… I especially had trouble controlling my anger. Lying in bed hurt, and moving also hurt. While everyone else was training, I was the only one who couldn’t move, which made me feel increasingly anxious. I ended up getting angry at my parents who were taking care of me, and then I felt guilty afterward.” (S001)*

*“What I felt most was anger. The doctor told me not to dance, and I was very angry that I couldn’t dance. I also felt disappointment and regret, and a sense of helplessness.” (S004)*

*“At that time, I was really angry with that teacher. I kept thinking, ‘Why did they push me like that when I was already in so much pain?’ We were doing dance sport, which doesn’t even require that much flexibility, so I couldn’t understand why it happened.” (S006)*

*“(My male partners) didn’t catch me. When that happened, my immediate thought was, ‘Why didn’t they catch me?’ I wondered how they could miss such a simple movement, and my first reaction was feeling a bit angry at them.” (S007)*

#### Theme 4. Guilt

Some participants reported feelings of guilt toward their parents and dance partners. Participant S003 felt guilty toward their parents, who accompanied them to the hospital and covered medical expenses following the injury. Similarly, participant S007 reported feeling guilty toward their dance partner, who was unable to participate in a competition due to the injury. These feelings of guilt extended beyond individual emotional responses and were understood within the cultural context of China, where familial responsibility and interdependence are highly valued. In particular, receiving support from others or perceiving that one’s condition negatively affected others appeared to intensify feelings of relational burden and psychological indebtedness.

*“While going through all the medical examinations, I felt very sorry when I saw my mother taking me from place to place. I kept thinking it would have been better if I hadn’t been injured. We also spent a lot of money, but I still didn’t recover.” (S003)*

*“For that competition, I had prepared a partner dance. My injury also affected my partner, so I felt somewhat guilty toward them. Because of me, they couldn’t perform in the partner piece, which made me feel very sorry.” (S007)*

#### Theme 5. Resilience

Although participants predominantly experienced negative emotions during injury rehabilitation, they nevertheless demonstrated a positive attitude toward the rehabilitation process. Through this process, all participants successfully completed rehabilitation and returned to dance, reflecting patterns of resilience.

*“‘It’s not a big deal,’ ‘If things don’t work out, I can try again next year,’ ‘I’ll definitely get better’—I kept telling myself these things, almost like self-hypnosis.” (S003)*

*“I kept trying to organize my thoughts and told myself not to focus only on negative things, but to think more generously. Gradually, my emotions shifted from confusion, irritation, and anxiety to a sense of calm. I became less restless and started to look forward to the day I would recover. I truly believed that I would get better.” (S005)*

*“Since my injury was mainly to my head, I tried to avoid overthinking. I did my best to control and regulate my emotions so I wouldn’t dwell on negative thoughts. I focused on resting as much as possible and preventing my brain from becoming overly active. I think this kind of emotional regulation really helped my recovery.” (S007)*

### Category 3. Social experiences

#### Theme 1. Emotional support

Participants reported receiving emotional support from their parents during injury rehabilitation. In particular, parents demonstrated an accepting attitude toward the various emotions and behaviors expressed by participants while coping with their injuries, and this support served as an important psychological resource. Participants also experienced emotional support from dance teachers and peers. The encouragement and reassurance provided by these individuals were reported to play a crucial role in sustaining engagement in the rehabilitation process and facilitating recovery.

“My family didn’t blame me, even when I got angry at them.” (S001)

“My parents never criticized me or did anything to hurt my feelings.” (S003)

“My parents kept comforting me, telling me to get plenty of rest and that it was okay not to attend classes.” (S005)

“After I got injured, my parents thought that injuries are common among dance students, but they never told me to stop dancing. They only suggested that I consider modifying certain movements. They continued to support and understand me.” (S007)

“My teacher told me that since I have good flexibility, I shouldn’t worry about falling behind others, and that they would help me catch up when I returned. That reassurance gave me a lot of comfort.” (S001)

“My dance teachers immediately asked about my condition. I reported my safety and recovery status to them every day. Whether it was my major instructor, academic advisor, or homeroom teacher, they were all very concerned about me.” (S007)

“My friends would buy me snacks I liked, and when I wasn’t at school, they would leave them on my bed. During video calls, they always shared positive stories and gave me a lot of encouragement and comfort. My online friends also supported me, but the emotional support from my close friends was the most meaningful.” (S001)

“What meant the most to me was the support from my friends. They told me that injuries are unavoidable in dance, and that they would support me whether I chose to continue dancing or to stop.” (S004)

#### Theme 2. Tangible support

Participants reported receiving treatment-related tangible support from their parents and teachers during injury rehabilitation. Medical expenses were primarily covered by parents, and both parents and teachers provided practical assistance related to hospital recommendations, appointment scheduling, and clinic visits. Some participants reported receiving guidance from teachers on rehabilitation exercises that facilitated recovery.

*“Financially, everything was covered by my family.” (S004)*

*“Every Saturday evening, our ballet teacher gave us stretching classes, which really helped with my injury.” (S004)*

*“My parents supported me in receiving rehabilitation training and physical therapy, including traditional treatments such as acupuncture and moxibustion.” (S005)*

“My teacher recommended places for physical therapy and suggested hospitals that had specialized treatment programs for lower back injuries.” (S006)

Participants also reported receiving tangible support from friends and parents during daily life. Peers who shared the same accommodation provided everyday assistance, such as helping with meals for participants experiencing physical discomfort. Similarly, parents supported participants by preparing preferred foods and assisting with daily activities.

*“My friends also took care of me. Since I was on the upper bunk and it was difficult for me to climb down, they brought me water and bought meals for me.” (S002)*

*“My friends bought meals for me, made sure I didn’t have to go down the stairs, and did everything they could to help me. They really took great care of me.” (S007)*

*“As for my parents, there’s no need to say much—during the month I stayed at home to recover, they cooked fish for me every day, and I gained about 5 kilograms.” (S007)*

#### Theme 3. Negative social experiences

Participants did not experience exclusively positive social interactions from those around them. Some reported criticism from parents and teachers regarding the circumstances of the injury and subsequent physical condition, which contributed to psychological distress. In some cases, teachers pressured participants to return early and resume training, which was perceived as burdensome. Furthermore, several participants reported experiencing indifference from teachers and peers after injury, leading to emotional distress.

*“My parents would sometimes say, ‘We told you not to learn dance back then, but you insisted, and now you’re injured.’ They didn’t say it often, but those words stayed with me and hurt me.” (S006)*

*“My friends and teacher said that I had gained weight from lying down, that it was very noticeable. My teacher even said, ‘Who would want to watch a pig dance?’ That comment still stays with me.” (S001)*

*“Some people also urged me to return to training quickly.” (S001)*

*“I had never taken such a long break before. Whenever I rested, my teacher would say, ‘Come back quickly, get back to class.”’ (S006)*

*“There were many students at the academy, so the teacher didn’t pay much attention to me.” (S004)*

*“Right after I got injured, they didn’t show me much concern or comfort. It was only after about a week that they did something. That really bothered me, because in the end, they were the ones who had put me in that situation.” (S007)*

The parents of participant S004 advised discontinuing dance following the injury. Although this recommendation was motivated by concern for the participant’s physical condition, it was experienced as emotionally distressing because the participant wished to continue dancing.

*“When my injury was confirmed, my mother immediately told me not to practice in front of the doctor, and the doctor also encouraged that if I stopped dancing, my back wouldn’t hurt. That made me feel upset, because I felt that my mother didn’t understand how I felt, even though I knew she said it out of concern.” (S004)*

## Discussion

This study explored the injury rehabilitation experiences of Chinese university dancers through an integrated biopsychosocial perspective. The findings indicate that dancers’ injury experiences are not limited to physical damage but constitute a complex process in which psychological responses and social environments are closely intertwined. Furthermore, certain psychological experiences, such as anxiety and guilt, were found to be embedded within the cultural value system specific to the Chinese context. These findings support the need for a multidimensional understanding of dance injuries as proposed by the biopsychosocial model (Engel, 1977), while also suggesting that this model should be interpreted within a culturally informed framework.

First, the biological experiences identified in this study, including pain, joint instability, reduced flexibility, and lower limb imbalance, were consistent with injury characteristics commonly reported in previous research on dancers. Muscle–tendon injuries, as well as low back and lower extremity injuries, accounted for a substantial proportion of dance-related injuries, consistent with previous studies. These injuries may be associated with repetitive movements and high-intensity training (Wei, 2026; Xu et al., 2025). Additionally, previously identified risk factors, including training intensity, technical inaccuracies in movement execution, and insufficient physical preparedness, were similarly reflected in the present findings (Zhu and Cao, 2025; Cheng and Wang, 2025, Campoy et al., 2011).

However, this study is distinguished by its interpretation of these physical injuries not merely as physiological problems, but within psychological and cultural contexts. In particular, somatic responses such as headaches, insomnia, and gastrointestinal symptoms demonstrated a close association with psychological stress, which is consistent with previous research highlighting the combined influence of physiological, psychological, and training-related factors (Cao et al., 2026b). Furthermore, such physical symptoms may be reinforced within the performance-oriented norms embedded in Chinese dance education, as well as within a cultural tendency toward the normalization of pain. This cultural context may lead dancers to perceive pain as an inherent part of training, potentially delaying early responses to injury and, consequently, contributing to the exacerbation of somatic symptoms.

Meanwhile, some participants described positive physical changes during rehabilitation, including increased muscle strength and improved flexibility, suggesting that injury rehabilitation may extend beyond simple recovery to a process of physical reconfiguration. This indicates that, when accompanied by systematic training adjustments and recovery strategies, injury may serve as a turning point that facilitates functional improvement. From a sport psychology perspective, these findings may be interpreted as resilience-related adaptation, whereby individuals develop positive physical and psychological changes through coping with injury-related challenges. Previous studies have suggested that athletes may experience resilience and positive growth following adversity through the development of greater self-awareness, adaptive coping strategies, confidence, and perceived social support (Galli and Vealey, 2008; Fletcher and Sarkar, 2012). Furthermore, the Sporting Resilience Model proposes that resilience in sport is a dynamic biopsychosocial process shaped through ongoing interactions between adversity, protective factors, and positive adaptation (Gupta and McCarthy, 2022). In the present study, some dancers appeared to reinterpret the rehabilitation process not only as recovery from injury but also as an opportunity to develop greater body awareness, movement control, and physical capacity.

In terms of psychological experiences, participants reported a range of negative emotions, including anxiety, frustration, anger, and guilt, while some also demonstrated resilience grounded in a positive mindset during injury rehabilitation. Anxiety about falling behind peers, as well as concerns regarding return to performance and the risk of reinjury, were closely linked to the competitive environment, reflecting the performance-oriented evaluation system within Chinese dance education.

The psychological responses experienced by injured dancers can be interpreted through the lens of Self-Determination Theory (SDT). According to SDT, an individual’s psychological wellbeing and motivation are determined by the fulfillment of three basic psychological needs: autonomy, competence, and relatedness (Deci and Ryan, 2000). In the present study, dancers experienced a decline in competence due to injury, while restrictions on training participation led to frustration of autonomy. Additionally, changes in relationships with peers and instructors posed a threat to relatedness. These experiences may contribute to reduced intrinsic motivation during injury rehabilitation. Accordingly, it is important to implement appropriate strategies to prevent declines in competence, autonomy, and relatedness among injured dancers throughout the rehabilitation process.

The emotion of guilt identified in this study warrants attention within the Chinese cultural context. This emotion is linked to collectivist values, in which personal setbacks are perceived as a burden on others, indicating that guilt extends beyond an individual feeling and is constructed within social relationships. Such negative psychological responses are consistent with previous findings suggesting that psychological factors play a significant role in both injury occurrence and the recovery process (Ardern et al., 2013; Pal et al., 2021; Xu et al., 2025).

In terms of social experiences, emotional support and tangible assistance functioned as important protective factors during injury rehabilitation. This finding reaffirms previous research indicating that social support is a key element in recovery (Yang et al., 2014; Forsdyke et al., 2022). At the same time, negative experiences, including pressure to return, indifference, and criticism, emerged as important risk factors that appeared to be closely associated with the hierarchical structure of Chinese dance education. In environments where instructor authority is particularly strong, dancers may be more likely to act in accordance with external expectations rather than their own physical condition, potentially increasing the risk of injury aggravation and reinjury. In the present study, interactions with parents, coaches, and peers were found to meaningfully influence the rehabilitation experiences of university dancers. In this regard, future studies may benefit from incorporating the perspectives of multiple stakeholders, such as parents, coaches, and dance partners, to provide a more comprehensive understanding of the biopsychosocial rehabilitation process.

In line with these findings, recent studies in China have emphasized the importance of multidimensional approaches to the prevention and rehabilitation of dance-related injuries. For example, an integrated intervention model combining physical capacity, technical skill, training load, and psychological factors has been proposed (Wei, 2026). In addition, training approaches grounded in exercise physiology and individualized rehabilitation strategies (Liu, 2025), as well as comprehensive risk management systems incorporating technique, environment, and recovery (Zhu and Cao, 2025), have been suggested. From the perspective of traditional Chinese medicine, the concept of “integration of medicine and dance” has also been introduced, aiming to combine therapeutic interventions with training in order to achieve physical recovery, functional maintenance, and aesthetic performance simultaneously (Cao et al., 2026a). These perspectives are consistent with the present findings, which highlight the integrated and interactive nature of biological, psychological, and social factors in injury rehabilitation.

A key finding of this study is that biological, psychological, and social factors do not operate independently but interact within a dynamic and cyclical structure to shape the injury rehabilitation experience. For example, physical pain may induce anxiety, which can hinder engagement in rehabilitation and ultimately lead to delayed physical recovery. Conversely, social support may serve a buffering role, mitigating this negative cycle. These findings support the importance of an integrated approach as emphasized in previous research on sports injuries (Brewer and Redmond, 2016).

Taken together, the injury rehabilitation experiences of Chinese university dancers reflected a dynamic interaction among biological injury, psychological responses, and social environments within a specific cultural context. These findings extend beyond the simple application of the biopsychosocial model and highlight the need for an expanded interpretive framework that incorporates cultural factors. In particular, the performance-oriented nature of Chinese dance education, its hierarchical structure, and its collectivist values were identified as key contextual elements shaping dancers’ perceptions of injury and rehabilitation experiences.

These findings may be interpreted within the framework of collectivist cultural values, in which maintaining group harmony and meeting social expectations are often prioritized over individual needs (Triandis, 2001). In addition, hierarchical relationships commonly observed in East Asian educational and performance settings may influence performers’ tendencies to comply with authority figures despite physical or psychological difficulties (Markus and Kitayama, 1991). Accordingly, future research should focus on developing integrated rehabilitation models that reflect these cultural and structural factors, and further expand both theoretical and practical understanding of dance injury rehabilitation through cross-cultural comparative studies.

This study included a relatively small sample of Chinese university dancers, which may limit the transferability of the findings to dancers from different cultural, educational, and athletic contexts. As this study aimed to provide an in-depth qualitative exploration rather than statistical generalization, the findings should be interpreted within the specific sociocultural context of Chinese university dance programs. Although sufficient data saturation was achieved, caution is needed when applying these findings to broader dancer populations worldwide. In addition, because this study relied on one-time interviews, it may not have fully captured the dynamic changes in rehabilitation experiences across different stages of recovery. Future longitudinal qualitative studies are needed to better understand how biopsychosocial experiences evolve over time.

## Conclusion

This study explored the injury rehabilitation experiences of Chinese university dancers through an integrated biopsychosocial perspective. The findings indicate that rehabilitation is not merely a process of physical recovery, but rather a complex and dynamic process in which physical symptoms, psychological responses, and social relationships interact, and that these experiences are shaped within a distinct cultural and structural context. The performance-oriented nature of Chinese dance education, its hierarchical relationships, and its collectivist value system were found to play a significant role in shaping how dancers perceive and cope with pain, as well as their psychological and social experiences during rehabilitation.

In addition, this study demonstrated that, alongside negative emotions and psychological difficulties, positive changes such as resilience and physical growth may also emerge during injury rehabilitation. This suggests that rehabilitation extends beyond recovery and can facilitate functional and psychological reconfiguration at the individual level. From a practical perspective, the findings indicate that interventions for dance injury rehabilitation should not be limited to physical treatment alone, but should adopt an integrated approach that includes psychological support and social environmental factors. In particular, the development of rehabilitation programs that incorporate cultural context may play a critical role in improving adherence to rehabilitation and recovery outcomes among dancers. Finally, this study is meaningful in that it provides an in-depth understanding of the injury rehabilitation experiences of Chinese university dancers through a qualitative approach. Future research should seek to expand this understanding through cross-cultural comparative studies and longitudinal designs, thereby contributing to a more comprehensive theoretical and practical understanding of dance injury rehabilitation.

## Data Availability

The datasets generated and/or analyzed during the current study are not publicly available due to the decision and recommendations of the Institutional Review Board of Korea National Sport University. However, the data are available from the corresponding author upon reasonable request.
